# Trivial chest trauma with incidentally detected radiographic findings

**DOI:** 10.4103/1817-1737.36557

**Published:** 2007

**Authors:** Suyash Mohan, Ashish Verma, Sunil Kumar

**Affiliations:** *Department of Radiology, Sanjay Gandhi Postgraduate Institute of Medical Sciences, Raebareily Road, Lucknow - 226 014, UP, India*

A 38-year-old woman presented with history of trivial chest trauma. A plain radiograph of chest was done for initial evaluation, which revealed a spectrum of incidental findings. On detailed retrospective history taking, the patient admitted of having breathlessness, worsening with physical activity; mucopurulent productive cough; and copious mucoid nasal discharge. She had recurrent sinusitis and had past history of recurrent chest infections, which were treated symptomatically from time to time. Her general physical examination was normal except for the fact that her normal heart sounds were heard on the right side of the chest. ENT examination showed deviated nasal septum (DNS) to the right.

## Image: CXR PA view, HRCT thorax and CT PNS

### Clinical questions

1. What are the radiological abnormalities?
2. What is the likely diagnosis?

### Imaging findings

**Figure 1:**
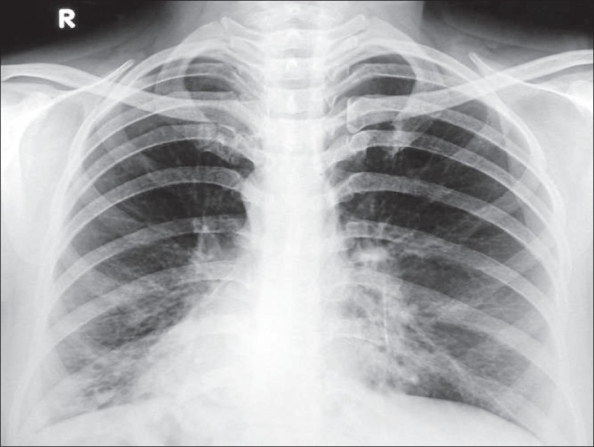
Chest radiograph PA view

**Figure 2:**
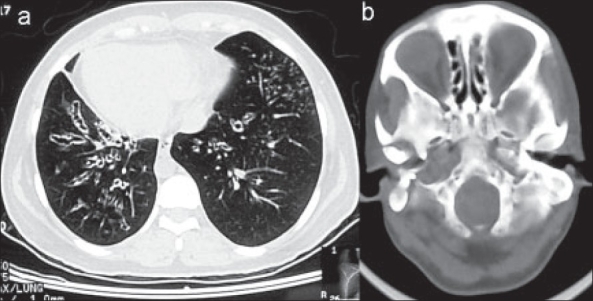
a) HRCT thorax. b) Axial CT PNS [bone window]

Chest X-ray [Figure 1] showed dextrocardia, which was supported by echocardiography. Multiple cystic changes were seen in the lower zones of both lung fields suggestive of bronchiectasis. High-resolution computerized tomography (HRCT) of the thorax was done, which confirmed the complex of situs inversus and cystic bronchiectasis seen on chest radiograph [Figure 2A]. CT of paranasal sinuses revealed DNS to the right and features of pansinusitis [Figure 2B]. Bronchoscopic examination showed bilateral mucopurulent secretion, predominantly in right lower lobe. ECG with reverse leads was normal. All other investigations were within normal limits. Presence of situs inversus, dextrocardia, bilateral cystic bronchiectasis and chronic sinusitis in combination confirmed the diagnosis of Kartagener's syndrome in this patient.

Situs inversus is a condition in which there is complete reversal of viscero-atrial situs and all the major organs are on the opposite side in the body as compared to their normal position. The presence of isomerism in addition implies the mirror image development of paired organs in the body - viz, right bronchial isomerism implies that both the bronchi are isomorphic to the right main bronchus. Primary ciliary dyskinesia (PCD) is a condition in which the cilia within organs, such as the nose, ears and lungs, do not function normally, with the result that mucus and bacteria are not cleared effectively causing chronic otitis media, rhinitis, sinusitis and recurrent chest infections. Bronchiectasis is a condition where there is abnormal dilatation of the airways.

## Discussion

Kartagener's syndrome is a congenital autosomal recessive disorder, a subgroup of immotile cilia syndrome. Incidence is 1 in 4,000–35,000 population,[1] accounting for one-tenth of the cases of bronchiectasis and about one-sixth of the cases of situs inversus.[2] Kartagener's syndrome is named after Kartagener who reported a series of cases with sinusitis, situs inversus including dextrocardia and bronchiectasis in 1933.[3] Male infertility was added to it in 1975.[3] Kartagener's syndrome may also be referred to as Kartagener's triad, Siewert's syndrome or Afzelius' syndrome. Kartagener's syndrome is a combination of PCD and situs inversus. If treatments are adhered to, life expectancy should be normal. As yet, there is no cure. The cilia of the reproductive organs are also abnormal and fertility is often reduced in patients with PCD. However, in the present case the fertility was unaffected and the patient had three full-term normal deliveries.
